# Spatial and Temporal Heterogeneities of *Aedes albopictus* Density in La Reunion Island: Rise and Weakness of Entomological Indices

**DOI:** 10.1371/journal.pone.0091170

**Published:** 2014-03-17

**Authors:** Sebastien Boyer, Coralie Foray, Jean-Sebastien Dehecq

**Affiliations:** 1 MIVEGEC, Institut de Recherche pour le Developpement (IRD) UMR 224, Centre National de la Recherche Scientifique (CNRS 5290), Universités Montpellier 1 and 2, Montpellier, France; 2 Centre de Recherche et de Veille sur les Maladies Emergentes dans l'Océan Indien (CRVOI), Sainte Clotilde, La Reunion Island, France; 3 Unité d'Entomologie Médicale, Institut Pasteur de Madagascar, Antananarivo, Madagascar; 4 GIP Service de prophylaxie renforcée, Service de Lutte antivecorielle, Agence Régionale de Santé de l'Océan Indien (ARS-OI), Saint Denis, La Reunion Island, France; Fondazione Bruno Kessler, Italy

## Abstract

Following the 2006 Chikungunya disease in La Reunion, questions were raised concerning the monitoring survey of *Aedes albopictus* populations and the entomological indexes used to evaluate population abundance. The objectives of the present study were to determine reliable productivity indexes using a quantitative method to improve entomological surveys and mosquito control measures on *Aedes albopictus*. Between 2007 and 2011, 4 intervention districts, 24 cities, 990 areas and over 850,000 houses were used to fulfil those objectives. Four indexes including the classical *Stegomyia* index (House Index, Container Index, Breteau Index) plus an Infested Receptacle Index were studied in order to determine whether temporal (year, month, week) and/or spatial (districts, cities, areas) heterogeneities existed. Temporal variations have been observed with an increase of *Ae. albopictus* population density over the years, and a seasonality effect with a highest population during the hot and wet season. Spatial clustering was observed at several scales with an important autocorrelation at the area scale. Moreover, the combination among these results and the breeding site productivity obtained during these 5 years allowed us to propose recommendations to monitor *Aedes albopictus* by eliminating not the most finding sites but the most productive ones. As the other strategies failed in La Reunion, this new approach should should work better.

## Introduction

The emergence of arboviruses and their vectors raised health and economic problems during the recent past years. The latest major arbovirus pandemic was due to the Chikungunya arbovirus which was not listed as a major health problem in Africa, Asia and to a lesser extent Europe. Chikungunya expansion started in Kenya in 2005, reached the South Western region of the Indian Ocean [1], [2] and in parallel Central Africa in 2005 [3], and then the European [4] and Asian continents [5]. The worldwide dispersal of this arbovirus was due to the lack of Human population immunity, and the efficient competence [6], ecology [7], dynamic [8] and density of the vectors [9]. The major vector incriminated during this pandemy was *Aedes albopictus* (Skuse) [10], [11]. The Chikungunya virus outbreak in 2005–2006 in La Reunion Island, located in the South West of the Indian Ocean, has initiated the monitoring of *Ae. albopictus* in urban areas of the Island by the vector control service of ARS (Agence Regionale de Sante), local representation of the French Ministry of Health.

*Ae. albopictus* is the major *Aedes* species in La Reunion since 1953 and its distribution can be observed in urban, suburban and natural areas [12]. The island landscape is divided by more than 300 ravines crossing urban environments, often composed of houses with gardens. Female *Ae. albopictus* can lay eggs in a high variety of natural and artificial breeding sites. In La Reunion, bamboo stump, rock holes, flower plates and small recipients are particularly attractive breeding sites [13]. In La Reunion, environmental conditions control the abundance of *Aedes albopictus* and adults generally emerge between 11 and 14 days after a rainy event. (Boyer et al. *In prep*). The meteorological data, the availability and the number of breeding sites, the dimension and the urbanization of the cities and the presence of green areas could affect the density of *Ae. albopictus* in the urban environment in La Reunion. Distribution, abundance, and individual fitness of the mosquitoes depend on biotic and abiotic factors [14], [15], [16], [17], i.e. the presence of unforested areas, water depth, and temperature. In addition, predators and competitors have also significant effects on mosquito larvae [18], [19]. Population abundance is markedly cyclical according to the season: the population of *Aedes albopictus* in New Jersey appeared to be very seasonal correlated with high daily temperature [20]. Moreover the productivity indexes in different urban breeding sites have been investigated on *Aedes aegypti* but few studies were carried on *Ae. albopictus*. A lack of information still exist on containers productivity and their relative contribution to the adult population size in the Island.

The indices used to evaluate *Stegomyia* population densities, such as the house index (HI: percentage of houses with at least one active breeding site), container index (CI: percentage of containers with larvae) and Breteau index (BI: number of active breeding sites per 100 houses) are widely used as empirical standard parameters [21]. These indices approximate the population size and determine monitoring threshold for control programs [11]. The Pan American Health Organization described 3 levels of risk for Dengue transmission with *Ae. aegypti* based on the HI (low (HI<0.1%), medium (0.1%<HI<5%), and high (HI>5%) [22]) and 2 risk levels for Yellow Fever transmission, with HI>1% and BI>5% [23]. These entomological indexes developed on *Ae. aegypti* can also allow to identify Dengue transmission areas [23]. The infested receptacle index (IRI: mean number of positive containers per house) was also used to measure public motivation in cleaning up their yards. Based on the strong correlation among the number of pupae and adults, pupae per person index [24], pupae per premises index (PPI) [21] and pupae per hectare index (PHI) [21] were used. In Italy, the number of laid eggs, adults and the pupal demographic monitoring were correlated [25]. But according to the vector control agency, neither pupal indexes or Adult Productivity Index (API) [26], can be daily or weekly monitored in La Reunion. However *Stegomyia* indexes failed to describe the risk of arbovirus transmission. For example, the Breteau index puts equal weight on all positive containers, however less than 20% of the total containers can be responsible for more than 80% of the *Aedes* adults [27], [28].

The biology of *Ae. albopictus* is related to water-filled containers distributed throughout suburban landscapes. Production levels of immatures were correlated with the abundance of mosquito-positive containers [29] and the amount of discarded containers in the habitat [30]. The distribution of the containers can create «hot spots» of mosquito production that can be point sources for reinfestation of neighborhoods [31]. The monitoring need to be made at the community level as a single non-complying household can infest an entire neighborhood [20]. In Peru, a clusterisation of the pupae was also related to *Ae. aegypti* dispersal [32]. Moreover, the mosquito production varied with containers' type in urban and suburban areas [29]. Based on the Index of Container Importance (ICI), a 60 increase-fold of the mosquito production was described due to the presence of positive containers in the neighborhood [29]. The occurrence of these hot spots may spatially vary over time, but the existence or absence of neighborhood clustering of *Ae. albopictus* can indicate locations where control efforts should be focused [29]. Indeed, the dispersal of *Ae. albopictus* is driven by the availability of oviposition sites [29]. More, the proximity of containers and other larval habitats increases the probability of pupae abundance [33]. The operational challenge is to adapt the better strategy thorough space and time [33].

We developed an approach with a geographic information system (GIS) combined with spatial statistics with temporal aspects. Indeed, vector control programs could be more efficient if the spatial locations of highly productive areas of *Ae. albopictus* can be predictable. The purpose of the present study was to identify *Ae. albopictus* breeding sites, to study the different productivity indexes and to improve the entomological survey monitoring. Without any a priori knowledge on the significance of each of these indexes on the *Ae. albopictus* population density, we will understand whether the density of mosquitoes is homogeneous or heterogeneous at a spatial and temporal scales.

## Materials and Methods

### Ethics statement

During one day randomly chosen per week, ARS agents asked for the permission to examine the water-holding containers in houses and checked every container. Residents provided informed consent to the agents to have their residence used in the study. In private properties, the owner gave permission to conduct the study on its site.The field studies did not involve endangered species. No study on vertebrate animals was carried out.

Additionally owners/residents gave permission to conduct a study on their land in order to determine the productivity of *Ae. albopictus* breeding sites. These visits occurred in a mission of Public Health and the agents from the Vector Control Service possessed annual District Ordinance allowing interventions in private places. In practice, the agents asked people to visit and it goes well in 98% of the cases with the people agreement. These visits represented around 70,000 visits per year. If people did not agree, the agents continued on to the next house unless the situation justified intervention with the municipal police (which was never the case for this research study). There is therefore no ethics committee since the Vector Control Service is not in research area but in a national mission of Public Health.

### Entomological survey and Productivity indexes

**Figure 1:**
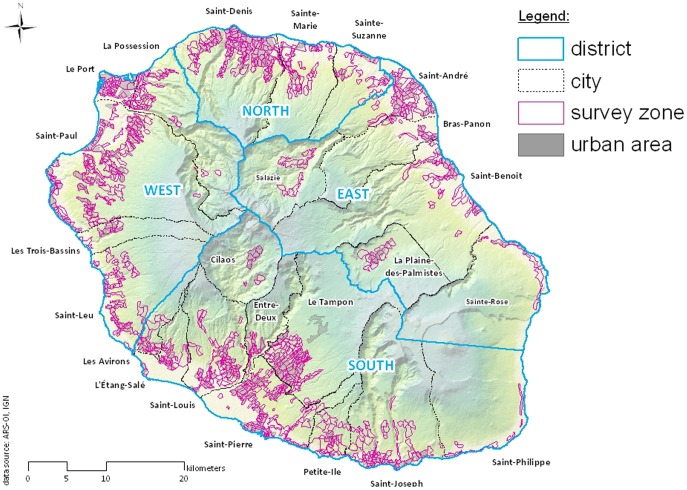
Map representing the 4 Mosquito Control Services (MCS) districts in La Reunion, the 24 towns and the 990 operational zones.

Vector control in La Reunion Island (21°10 S; 55°30 E) is divided into 4 districts grouping 24 towns (Figure 1). Urban areas were divided into 990 operational zones where the breeding sites were observed and systematically destroyed or treated. One zone had 40 up to 711 houses with an average of 190 houses per area (median = 170 houses per area). Four to 10 sites were visited every day between January 2007 and December 2011. No *Ae. albopictus* monitoring existed prior to 2006 Chikungunya outbreak.

The inspection of natural and artificial breeding sites were carried out in urban and rural areas to identify and destroy breeding sites, and also to inform people about prophylaxis and vector diseases. Traditional *Stegomyia* indexes (House Index HI, Container Index CI, Breteau Index BI) and Infested Receptacle Index (IRI) have been selected from this entomological study (Table 1). Public spaces such as schools and parks were also monitored regularly.

**Table 1:** Definition of the productivity indexes.

Name of Index		Definitions
House Index	HI	Percentage of houses infested with larvae and/or pupae
Container Index	CI	Percentage of water-holding containers infested with active immatures
Breteau Index	BI	Number of positive containers per 100 houses
Infested Recipient Index	IRI	Number of positive containers per houses infested with larvae and/or pupae

The pupae per premises index (PPI), pupae per hectare index (PHI) and the adult density index (ADI) were not used during the monitoring. From a practical point of view, *Culex* and *Aedes* pupae are present in the same breeding sites in La Reunion, and it is not possible for the agents to distinguish easily and rapidly the different species. The PHI was also not used because it existed an important heterogeneity inside the same city or zone in term of human population density and habitat density. Moreover, the following of the habitat study was not possible in term of agent complement.

### Monitoring of breeding sites

During the routine service request inspection process, breeding sites were destroyed and larvicide was applied only rarely. Yard sanitation and education of the residents are also part of the service request routine: agents demonstrated how to find and definitively eliminate mosquitoes' breeding sites and explained the part of each owner in the integrated community-based control strategy for preventing La Réunion Island from outbreaks. The agents also give some advices in case of active arbovirus transmission for the protection against mosquito bites (use of repellents, trousers and long sleeves, and see quickly a doctor in a case of fever).

Between 2007 and 2011, 60 houses per zone were controlled. Every container containing water was recorded in both the ravines and the urban areas every week. First, immature stages were collected with a pipette and the number of larvae (3^rd^ and 4^th^ instars), pupae were counted, and productivity (pupae+larvae) was calculated. Every single container with water was described (physical characteristic: size, depth, amount of water, water quality and appearance (clear, tinted, turbid, polluted), and shade (shadow, mi-shadow, sunny).

**Figure 2:**
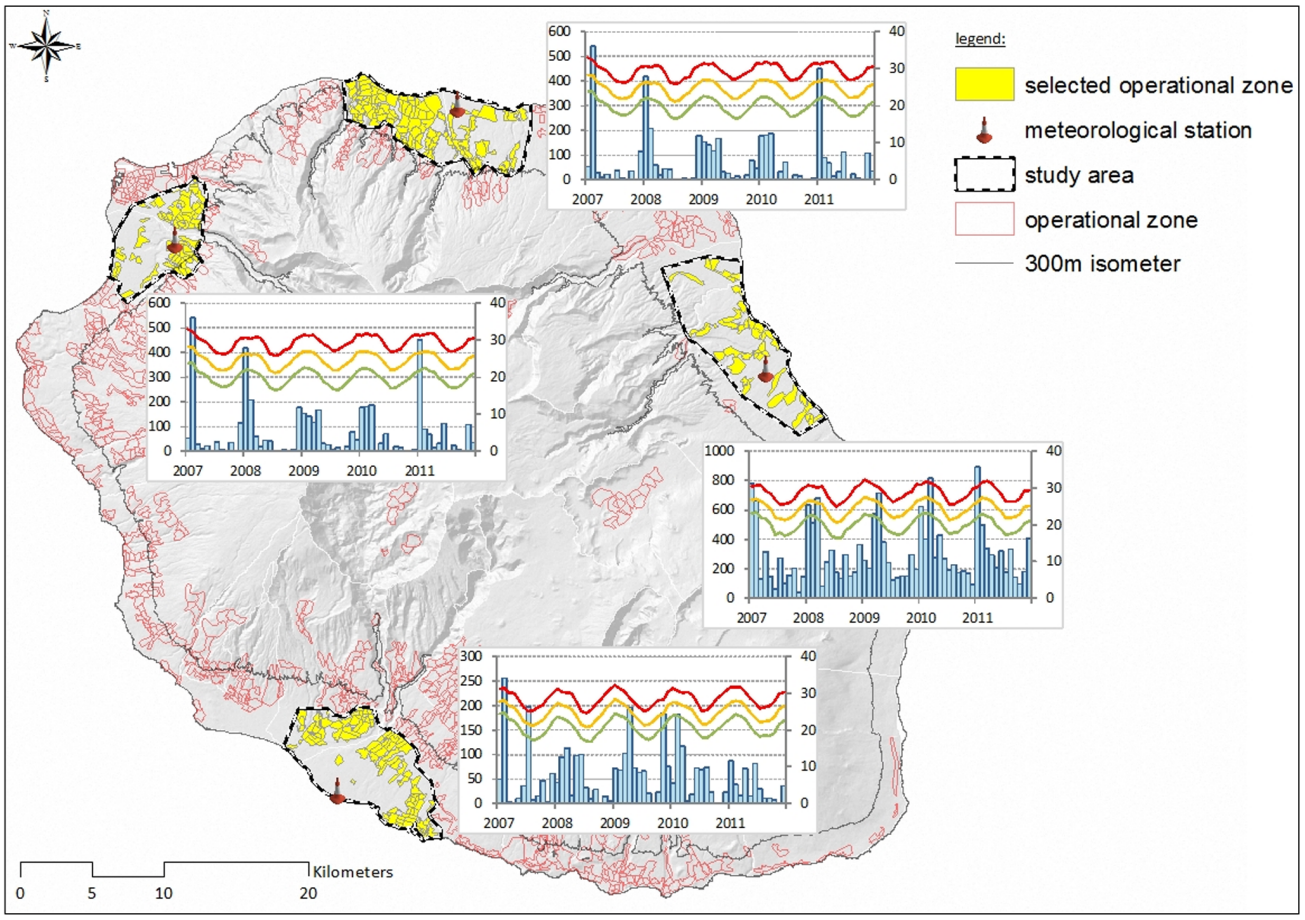
Map representing the 4 different zones choose for the meteorological analysis, and the representations of the average precipitation per zone over a year and the minimum, average and maximum temperature.

Four meteorological stations in four cities (Saint-Pierre, Saint-Denis, Saint-Paul, Saint-Benoît) in the 4 different districts were chosen to determine the effect of precipitations and temperature on the index variation. The zones were chosen near to the meteorological stations (less than 2 kms) with an altitude reflecting the altitude of the meteorological station (less than 300 m) and with a continuous coherent landscape (Figure 2).

### Weekly monitoring

A weekly monitoring was decided to estimate the natural variation within homogeneous breeding sites. One site was followed during 32 weeks in order to estimate productivity fluctuations. Nine different bamboo holes, as a natural habitat, were followed at the same place with the same environmental and meteorological conditions (same temperature, precipitations, relative humidity, wind). Bamboo holes represented one of the most frequent *Ae. albopictus* natural breeding sites encountered in La Reunion: identical bamboo stumps (same size, same depth) spaced out 50 cms and located in the ravine “Chemin Severe” in Saint-Benoit were chosen. Each bamboo stump was emptied every week and larvae and pupae were counted and then returned to the hole. Natural water was supplied every week.

### Data analysis

All data were exported and carried out with JMP 8.0 (Statsoft inc., Paris, France), R 2.14.1 freeware (The R Development Core Team, 2008) and ArcGIS 10.0 (GIS software: ESRI ArcGIS™, Redlands, CA, United States).

#### Spatial analysis

The spatial analyses were carried out on the same dataset of 6548 zones used during the temporal analyses. Analyses were carried out on the four indexes per year. We used the global Moran index to test the spatial autocorrelation choice distance (ArcGis Analyst Spatial Tools; 500, 800 and 1000 meters were tested). The Moran's coefficient of autocorrelation test determines whether the distribution of these indexes is spatially clustered, dispersed or random in space [34]. The location of clusters and outliers was detected with the local Moran test [35] (ArcGIS, Spatial Analyst Tools). LISA (Local Indicators of Spatial Association) identified the core of a cluster analysing the values of sites per pair. A local Moran index was determined for each site. The sum of all the local indices was observed proportionality with the global Moran index, which take into account the average value. Then, local Moran indices were compared to the global Moran index in order to determine the nature of the spatial liaison (High-High, High-Low, Low-High or Low-Low). The significance was calculated after a normalization of the local Moran indices (P = 0.05). Analyses were parameterized with the neighbour sites in fixed distance band of 1000 meters. Map representations were specifically developed using the built-in GIS software functions (Arc-Info GIS 9.3 grid module, ESRI Inc).

#### Meteorological analysis

The meteorological analyses were carried out on a dataset of 957 data in the North (Saint-Denis), 457 in the South (Saint-Pierre), 329 in the West (Saint-Paul) and 501 in the East (Saint-Benoît). The step function results allowed to create nested models. We chose the best model with effecting likelihood-ratio test. After, a Pearson test was performed to determine whether the model was correctly adjusted. The residuals were also analyzed to make sure that normality and homoscedasticity of residuals were followed to carry out an ANOVA.

#### Temporal analysis

Meteorological data were used to make a time series by day-decades for Saint-Denis zona (The 3.88% missing data were extrapolated from the average of the previous and next indice's values). The stationary (Dickey Fuller test) and the autocorrelation (Ljung-Box test) were tested for each index. The time series were divided into three parts (general, seasonal, residuals). Then a global linear model was carried out with the meteorological data to explain the seasonal results the residuals.

#### Productivity analysis

Analyses were performed on a dataset coming from 1323 breeding sites to study the effect of environmental factors on the productivity. The differences among the types of the container were indicated by an ANOVA. The correlations between the numbers of immature *Ae. albopictus* and 9 factors (width, depth, type of the breeding site, the town, the organic matter, the effect of the sunshine, the presence of *Anopheles* and *Culex* and the quality of the water) were tested with Pearson tests. The influence of the significant factors on the presence of immature stages has been tested using an ANOVA.

## Results

850,804 houses, equally distributed among the four districts and representing 25% of the total houses, were visited (Table 2). However, 50.5% of these houses were not inspected due to the absence of the inhabitants or the decline of the owners. 16.6% of houses were positive, representing 69,958 positive houses with 148,741 positive containers (Table 2).

**Table 2:** Number of houses visited in 2007–2011 in the 24 towns in La Reunion Island.

District	City	Visited houses	Inspected houses	Positive houses	Positive containers
		(% of total houses)	(% of visited houses)	(% of inspected houses)	
*North*		*209347 (24.6%)*	*91285 (43.6%)*	*16400 (18.0%)*	*37925*
	Saint-Denis	129390 (15.2%)	54427 (42.1%)	9181 (16.9%)	21594
	Sainte-Suzanne	32898 (3.9%)	15713 (47.8%)	3146 (20.0%)	6869
	Sainte-Marie	47059 (5.6%)	21145 (44.9%)	4073 (19.3%)	9462
*South*		*236871 (27.8%)*	*122203 (51.6%)*	*21749 (17.8%)*	*45469*
	Saint-Pierre	51974 (6.1%)	26061 (50.1%)	4777 (18.3%)	10410
	Le Tampon	49159 (5.8%)	24341 (49.5%)	4230 (17.4%)	8975
	Saint-Louis	46199 (5.4%)	24197 (52.4%)	4244 (17.5%)	8609
	L'Etang-Sale	14693 (1.7%)	7781 (53.0%)	1294 (16.6%)	2646
	Saint-Joseph	31618 (3.7%)	17036 (53.9%)	3312 (19.4%)	6858
	Petite Ile	13360 (1.6%)	6999 (52.4%)	1246 (17.8%)	2628
	Les Avirons	13110 (1.6%)	6820 (52.0%)	1074 (15.7%)	2013
	Saint-Philippe	6054 (0.7%)	3574 (59.0%)	754 (21.1%)	1658
	L'Entre Deux	8734 (1.0%)	4530 (51.9%)	763 (16.8%)	1581
	Cilaos	1970 (0.2%)	864 (43.9%)	55 (6.4%)	91
*East*		*194684 (22.9%)*	*98131 (50.4%)*	*16235 (16.5%)*	*35393*
	Saint-Benoit	57682 (6.8%)	30122 (52.2%)	5213 (17.3%)	11838
	Bras-Panon	19068 (2.2%)	9828 (51.5%)	1728 (17.6%)	3753
	Salazie	12878 (1.5%)	7114 (55.2%)	854 (12.0%)	1910
	Plaine des Palmistes	14630 (1.7%)	5672 (38.8%)	444 (7.8%)	867
	Saint-Andre	77340 (9.1%)	37825 (48.9%)	6491 (17.2%)	13578
	Sainte-Rose	13086 (1.6%)	7570 (57.8%)	1505 (19.9%)	3447
West		*209902 (24.7%)*	*109098 (52.0%)*	*15574 (14.3%)*	*29954*
	Saint-Paul	104509 (12.3%)	53572 (51.3%)	7722 (14.4%)	14926
	La Possession	31434 (3.7%)	15331 (48.8%)	2166 (14.1%)	4323
	Le Port	27388 (3.2%)	15455 (56.4%)	2097 (13.6%)	3787
	Trois Bassins	10585 (1.2%)	5747 (54.3%)	748 (13.0%)	1595
	Saint-Leu	35986 (4.2%)	18993 (52.8%)	2841 (15.0%)	5323
Total		850804	420717 (49.5%)	69958 (16.6%)	148741

The number of visited and positive houses and the number of positive containers have been detailed.

### Temporal analysis

**Figure 3:**
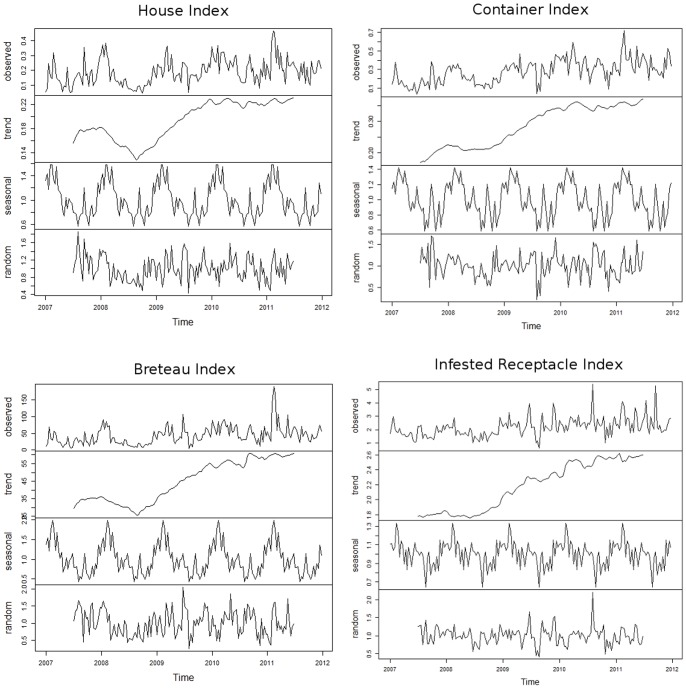
Variation of the four different index from 2007 up to 2011. The time series were divided into three parts: the general trend (trend), the seasonal results (seasonal), and the residuals (random).

Temporal analysis divided the real values into three different parts, i.e. the global trend of the considered index, the seasonality of the index and the residuals (Figure 3). In general the indexes increased from 2007 to 2010, reaching a plateau after 2010. In details, House Index (HI) values oscillated between 0.14 and 0.18 before 2009, from 0.14 to 0.22 during 2009 and reached a plateau at the beginning of 2010. Breteau Index (BI) values presented the same trend with values oscillating between 25 and 35 before 2009, from 25 up to 55 in 2009 and 2010 and reached a plateau at the beginning of 2011. Ci and IRI values increased continuously from 0.18 up to 0.36 and from 1.8 up to 2.5, respectively. A significant seasonality was observed with the four indices: An annual cycle was apparent in all the zones with low index values during the winter (dry and cold) and high index values during the summer (humid and hot), but with substantial heterogeneity in the relative strength of the multiannual cycle, especially the timing of annual picks. In details, HI and CI values were high from December to February, and low from April to November for HI and between July and November with BI, with a only high pick in the middle of August. BI values were high in January and February and low from May to November. The seasonality of IRI was significant but the seasonality was less marked than the other indices. The residuals were tested and correlated with climatic factors including the precipitations and the temperatures (Table 3).

**Table 3:** Correlation between the meteorological analyses and the seasonal and random trends of the temporal analyses.

HOUSE INDEX			CONTAINER INDEX			BRETEAU INDEX			INFESTED RECEPTACLE INDEX		
*Augmented Dickey-Fuller Test*			*Augmented Dickey-Fuller Test*			*Augmented Dickey-Fuller Test*			*Augmented Dickey-Fuller Test*		
Dickey-Fuller	Lag order	p-value	Dickey-Fuller	Lag order	p-value	Dickey-Fuller	Lag order	p-value	Dickey-Fuller	Lag order	p-value
−3.846	5	0.01825	−4.469	5	0.01	−4.4531	5	0.01	−5.0588	5	0.01
Alternative hypothesis: stationary			Alternative hypothesis: stationary			Alternative hypothesis: stationary			Alternative hypothesis: stationary		
*Box-Ljung test (autocorrelation)*			*Box-Ljung test (autocorrelation)*			*Box-Ljung test (autocorrelation)*			*Box-Ljung test (autocorrelation)*		
X-squared	df	p-value	X-squared	df	p-value	X-squared	df	p-value	X-squared	df	p-value
80.5375	1	<2.2e-16	84.1494	1	<2.2e-16	64.6353	1	8.88E-16	30.9535	1	2.64E-08
*Seasonal Trend (Gaussian Model)*			*Seasonal Trend (Gaussian Model)*			*Seasonal Trend (Gaussian Model)*			*Seasonal Trend (Gaussian Model)*		
P0; P1; P2; Tmin; P1 × P2; P2 × Tmoy;			P0; P1; P2; Tmin; P2 × Tmoy; Tmin × Tmoy			P0; P1; P2; Tmin; P1 × P2; P2 × Tmoy;			P0; P1; P2; Tmin; Tmin × Tmoy; P2 × Tmax		
Tmin × Tmoy; Tmin × Tmoy × Tmax			P1 × P2 × Tmoy; Tmin × Tmoy × Tmax			Tmin × Tmoy; Tmin × Tmoy × Tmax			P1 × P2 × Tmoy; P1 × Tmin × Tmoy;		
									Tmin × Tmoy × Tmax		
*Random Trend (Gaussian Anova)*			*Random Trend (Gaussian Anova)*			*Random Trend (Gaussian Anova)*			*Random Trend (Gaussian Anova)*		
P1; Tmoy; Tmax; P1 × Tmin; P0 × P2 × Tmin			Tmoy			P1; Tmoy; P0 × Tmin; P0 × P2 × Tmin			T moy; P0 × Tmin; Tmoy × Tmax		

The stationary (Dickey Fuller test of null hypothesis of temporal non stationary) and the autocorrelation (Ljung-Box test) were tested for each index. p0 represents the rain precipitations during the decades the measures have been done, p1 during the previous decade, and p2 during the decade before. Tmin, Tmoy and Tmax represent respectively the weekly minimum, average and maximum temperatures.

One site containing identical bamboo stumps breeding sites was followed up every week during 32 weeks in order to estimate the weekly productivity variation. 6,015 larvae and 492 pupae were counted varying from 14 up to 183 larvae and from 0 to 32 pupae depending on the date and the breeding site. Significant effects of the breeding site factor were observed on the number of larvae (Df = 2; F = 17.43; P<0.0001), pupae (Df = 2; F = 14.41; P<0.0001) and the number of the total immatures (Df = 2; F = 19.04; P<0.0001), but not on the ratio pupae/larvae (Df = 2; F = 1.66; P = 0.19). The ratio among the minimum and the maximum immature number was 2.48 ± 0.45 in average.

### Meteorological analysis

The BI cannot be explained by the meteorological factors: the model cannot be adjusted, meaning that other factor(s) should explain this index. The interactions between the temperatures (minimum, maximum or average) and the precipitations had significant influences on HI, CI and IRI (Table 4). The geographical differences into temperature and humidity between the different districts (Figure 2) did not affect these correlations. Indeed, depending on the considered district and index, all the variables interacted and no particularly meteorological factor could be highlighted.

**Table 4:** Correlations between the meteorological analyses and 3 indexes.

Meteorological variables	North			South			East			West		
	CI	HI	IRI	CI	HI	IRI	CI	HI	IRI	CI	HI	IRI
p0	**0.002**	**<0.0001**	**0.004**	0.722	**<0.0001**	0.390	**<0.0001**	**<0.0001**	**0.001**	0.981	0.073	0.762
p1	**<0.0001**	**<0.0001**	**<0.0001**	**0.026**	**<0.0001**	0.501	**<0.0001**	**<0.0001**	**0.001**	0.282	0.257	0.136
p2	**0.047**	0.003	0.945	0.052	**<0.0001**	0.181	0.513	**0.026**	0.669	0.310	0.022	0.390
Tmin	**<0.0001**	**<0.0001**	**0.000**	0.055	0.314	0.149	**0.002**	**0.001**	0.389	**<0.0001**	**<0.0001**	**0.003**
Tmoy	0.126	0.135	0.396	0.051	0.000	0.193	**0.038**	0.249	0.816	**<0.0001**	**<0.0001**	**<0.0001**
Tmax	**0.001**	0.780	0.574	0.060	0.958	0.318	**0.0003**	**0.003**	0.486	**0.001**	**0.001**	0.255
p0:p1	0.541	0.619	0.533	0.856	0.549	0.595	0.070	**0.025**	0.206	**0.000**	**0.025**	**0.006**
p0:p2	**0.000**	**<0.0001**	0.121	0.933	0.999	0.762	0.813	0.965	0.673	0.470	0.771	0.666
p1:p2	**<0.0001**	**<0.0001**	0.043	0.117	0.050	0.778	0.086	**0.036**	0.659	**0.038**	**0.041**	0.121
p0:Tmin	**0.001**	0.322	**0.0004**	0.167	0.721	0.640	0.068	0.328	**0.0001**	0.670	0.447	0.846
p1:Tmin	0.058	**0.006**	0.965	**0.0001**	**0.002**	**0.006**	0.062	0.574	0.071	0.114	0.062	0.725
p2:Tmin	**0.001**	**0.020**	0.266	0.097	0.118	0.362	0.119	0.850	0.854	0.820	0.842	0.412
p0:Tmoy	0.075	0.818	0.319	0.107	0.056	0.799	**0.001**	0.244	0.608	0.688	**0.003**	0.073
p1:Tmoy	0.251	**0.001**	0.674	0.423	0.587	0.720	0.944	0.252	0.103	0.150	0.081	0.060
p2:Tmoy	0.078	0.239	0.760	0.163	0.115	0.657	0.524	0.226	0.259	0.622	0.086	0.671
Tmin:Tmoy	**0.037**	0.207	0.520	**0.0002**	0.237	0.064	0.166	0.558	**0.003**	0.725	0.685	0.359
p0:Tmax	0.299	0.505	0.784	**0.000**	0.054	0.304	0.936	0.549	0.033	0.486	0.168	0.538
p1:Tmax	0.820	0.772	0.175	0.897	0.066	0.646	0.390	0.401	0.140	**0.010**	0.112	0.195
p2:Tmax	0.090	0.864	0.519	0.068	0.667	0.416	0.990	0.568	0.881	**0.012**	**0.047**	0.556
Tmin:Tmax	**0.000**	**<0.0001**	0.093	**0.003**	**0.000**	0.496	0.285	0.027	0.318	0.285	0.613	0.308
Tmoy:Tmax	0.340	0.420	0.299	0.130	0.350	0.161	0.102	0.623	0.070	**<0.0001**	**0.007**	**0.001**
p0:p1:p2	0.241	0.284		0.454	0.534	0.894	**0.016**	0.334	0.385	0.144	**0.029**	0.064
p0:p1:Tmin	**0.000**	**0.008**		**<0.0001**	**<0.0001**	0.218	0.875	0.989	0.272	**0.003**	**0.031**	**0.036**
p0:p2:Tmin	0.589	0.054		0.923	0.081	0.207	**0.002**	0.354	**0.009**	0.755	0.538	0.901
p1:p2:Tmin	0.073	0.147		**0.040**	0.055	0.212	**0.024**	0.715	0.605	0.517	0.469	0.930
p0:p1:Tmoy	0.416	0.373		**0.016**	**0.029**	0.311	0.440	0.103	0.398	0.276	0.436	**0.012**
p0:p2:Tmoy	0.622	0.102		0.130	0.249	0.267	0.693	0.351	0.670	0.201	**0.005**	0.102
p1:p2:Tmoy		0.260		0.962	0.431	0.147	0.168	0.554	0.406	**0.000**	**0.005**	0.145
p0:Tmin:Tmoy	0.753	0.670		0.301	0.578	0.957	0.691	0.628	0.193	0.382	0.066	0.679
p1:Tmin:Tmoy	**0.026**	**0.0001**		0.512	0.914	0.706	0.169	0.684	0.622	0.635	0.192	0.154
p2:Tmin:Tmoy	0.588	0.815		0.303	0.165	0.318	0.018	0.269		0.364	0.754	0.785
p0:p1:Tmax	0.510	0.154		**0.003**	**0.003**	**0.033**	0.664	0.073	0.673	0.345	**0.044**	0.104
p0:p2:Tmax	0.639	0.425		0.266	0.256	0.625	0.393	0.207	0.427	0.789	0.908	0.881
p1:p2:Tmax	**<0.0001**	0.481		**0.016**	0.207	0.329	0.157	0.539	0.580	0.584	0.064	**0.038**
p0:Tmin:Tmax	0.959	0.975		0.105	0.096	0.455	0.592	0.646	0.200	**0.001**	0.166	**0.002**
p1:Tmin:Tmax	0.040	0.136		0.358	0.854	0.552	0.204	0.243	**0.0002**	0.915	0.417	0.831
p2:Tmin:Tmax	0.676	0.191		0.887	0.923	0.999	0.111	0.742	0.500	0.087	0.057	0.171
p0:Tmoy:Tmax	0.164	**0.012**		0.266	0.407	0.772	0.949	0.487	0.151	0.253	0.855	0.072
p1:Tmoy:Tmax	0.433	0.807		0.211	0.905	0.330	0.182	0.268	0.241	0.179	0.166	0.772
p2:Tmoy:Tmax	0.873	0.068		0.195	0.441	0.514	**0.042**	0.194	0.425	0.413	0.032	0.380
Tmin:Tmoy:Tmax	**0.007**	0.367		**0.032**	0.481	0.212	0.080	0.895	0.358	0.141	0.112	0.544

The analyses were carried out on a dataset of 957 data in the North (Saint-Denis), 457 in the South (Saint-Pierre), 329 in the West (Saint-Paul) and 501 in the East (Saint-Benoît). CI represent Container Index, HI House Index, IRI Infested Receptacle Index. p0 represents the rain precipitations during the decades the measures have been done, p1 during the previous decade, and p2 during the decade before. Tmin, Tmoy and Tmax represent respectively the weekly minimum, average and maximum temperatures. Bold numbers represents significant correlations. Grey cells represent cases with non adjusted model.

### Spatial analysis

Significant differences of index values were observed among the four districts (CI: Df = 3, F = 2.89, P = 0.034; HI: Df = 3, F = 47.45, P<0.0001; BI: Df = 3, F = 50.22, P<0.0001; IRI: Df = 3, F = 28.03, P<0.0001) with highest values obtained in the South and in the North and the lowest values in the West. At a smaller scale, significant differences among the 24 cities were observed. The highest index values were observed in Saint-Pierre, Saint-Philippe, Sainte-Suzanne, Sainte-Marie and Saint-Benoît. The lowest values were observed in Salazie, Cilaos, Plaine des Palmistes and Le Port.

**Figure 4:**
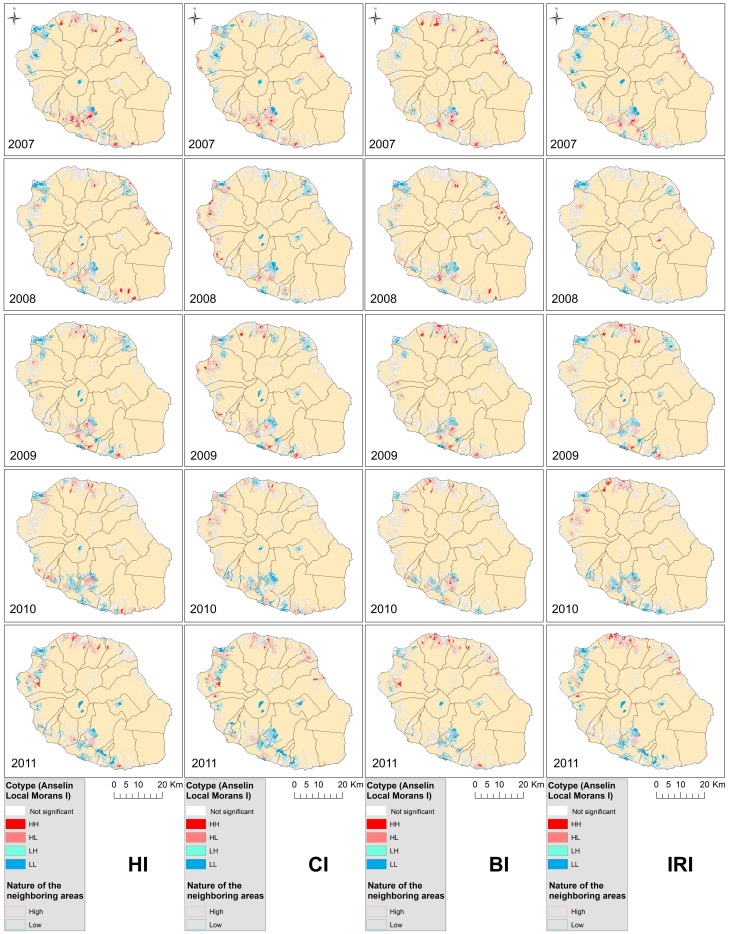
Variation of the clusterisation for each index depending of the considered year. This clusterisation is the Anselin representation of the clusterisation of local Moran index. HH represents High-High clusters which is a cluster with a high index surrounded with clusters with high index; LL Low-Low, LH Low-High, and HL High-Low.

The analysis of the 990 zones showed a spatial clusterisation of the density of *Ae. albopictus* in 2007, 2010and 2011 (Table 5). In 2008, CI is the only clustered index whereas in 2009 all the indices were clustered except HI (Table 3). Variations of the clusterisation also existed for each index depending of the year (Figure 4). For example, we can observe a majority of ‘Low-Low’ clusters in Saint-Paul in 2007 indicating a low density of mosquito, but a majority of ‘High-High’ clusters in 2008 indicating an important variation in the same city depending of the year. This pattern of index value over time can be observed in several zones: only Cilaos, Salazie and Plaine-des-Palmistes conserved clusters with low levels or no clusterisation.

**Table 5:** Spatial analysis of the 4 indexes, based on a dataset of 6548 zones.

Year	Index	Distance = 500 m		Distance = 800 m		Distance = 1000 m		Interpretation
		Morans Index	P-value	Morans Index	P-value	Morans Index	P-value	
2007	CI	0.005	0.904	0.075	**0.012**	0.072	**0.003**	Clustered (800 m)
	HI	0.208	**0.0001**	0.239	**<0.0001**	0.225	**<0.0001**	Clustered (500 m)
	BI	0.092	0.081	0.109	**0.0002**	0.127	**<0.0001**	Clustered (800 m)
	IRI	0.138	0.779	0.109	**0.0003**	0.075	**0.003**	Clustered (800 m)
2008	CI	0.641	0.211	0.082	**0.004**	0.084	**0.0003**	Clustered (800 m)
	HI	0.034	0.496	0.032	0.242	0.041	0.070	Random
	BI	0.015	0.76	0.035	0.204	0.044	0.051	Random
	IRI	−0.002	0.976	0.003	0.844	0.009	0.574	Random
2009	CI	0.071	0.163	0.077	**0.008**	0.107	**<0.0001**	Clustered (800 m)
	HI	−0.011	0.855	0.029	0.292	0.041	0.078	Random
	BI	0.005	0.902	0.045	0.116	0.063	**0.006**	Clustered (1000 m)
	IRI	−0.026	0.63	0.013	0.605	0.024	0.276861	Random
2010	CI	0.018	0.71	0.091	**0.002**	0.052	**0.019**	Clustered (800 m)
	HI	0.130	**0.011**	0.187	**<0.0001**	0.146	**<0.0001**	Clustered (500 m)
	BI	0.050	0.318	0.124	**<0.0001**	0.102	**<0.0001**	Clustered (800 m)
	IRI	0.007	0.868	0.084	**0.004**	0.081	**0.0005**	Clustered (800 m)
2011	CI	0.026	0.575	0.109	**0.0001**	0.090	**<0.0001**	Clustered (800 m)
	HI	0.097	**0.044**	0.137	**<0.0001**	0.114	**<0.0001**	Clustered (500 m)
	BI	0.134	**0.005**	0.181	**<0.0001**	0.126	**<0.0001**	Clustered (500 m)
	IRI	0.050	0.293	0.102	**0.0002**	0.093	**<0.0001**	Clustered (800 m)

A global Moran index tested the spatial autocorrelation choice distance at 500, 800 and 1000 m. The P-value was calculated after a normalisation of the local Moran indices (in bold, P<0.05).

### Breeding site productivity

1,948 breeding sites have been assessed with 1,323 positive breeding sites (67.9%) in which 33,332 larvae and 4,554 pupae were counted. The number of pupae, larvae (instar 3 & 4) and the productivity (pupae + larvae) were correlated with length, diameter, sun exposition, types of breeding sites, and depth of the breeding sites. Moreover, the number of pupae was correlated with the quality of the water. These 3 variables were not correlated with the area, cities or with the presence/absence of *Anopheles* and *Culex* individuals. In details, the number of *Aedes* was significantly higher in the deepest and widest shaded breeding sites. In the same way, more larvae and pupae were found in the largest surface breeding-sites (observed maximum area = 8 m×4 m). The water quality was correlated only with the number of pupae: more pupae were counted in troubled and polluted water than in clear water.

The productivity was significantly higher in the breeding sites constantly under shadow (60.10) than in the breeding sites always exposed to the sun (5.43). The breeding sites alternatively under the shadow or exposed to the sun presented an intermediate productivity (22.54). The same pattern was observed with larvae and the pupae.

**Figure 5:**
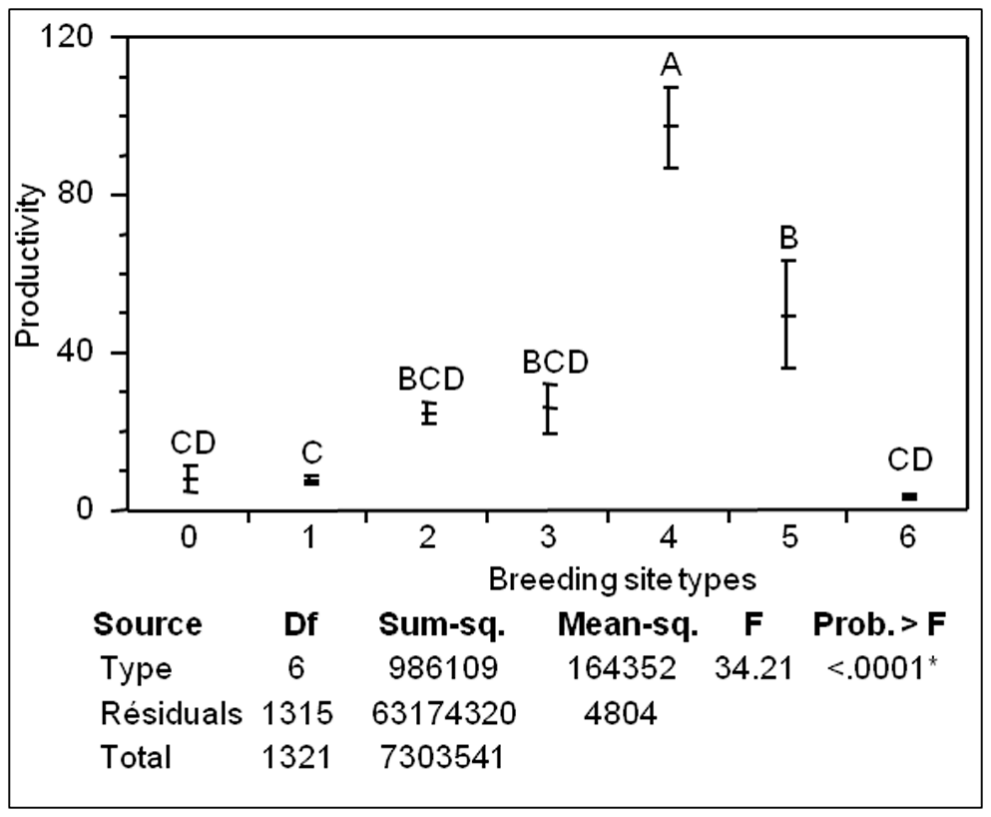
Productivity of breeding site of *Aedes albopictus* (N = 1949 observations). Natural breeding sites (0), Flower plates (1), Small recipients (2), Big recipients (3), Tires (4), Basin and reservoir (5) and vegetal breeding sites (6). Different letters mean that the mean values of productivity (average of larvae and pupae in a breeding site) are statistically different (Tukey's t test; P<0.05).

*Ae. albopictus* breeding sites were classified into 7 classes: natural breeding sites such as bamboo stumps, tree and rock holes generally found in ravines, flower plates, small and big containers, tires, basins and reservoirs, and vegetal ones. The number of immature *Ae. albopictus* per breeding sites ranged from 5.3 up to 26.8 in natural breeding sites, vegetal breeding sites and flower plates. Intermediate productivity values were obtained in small and big containers (71.5–72.5) (Figure 5). The highest numbers of immature were found in basins and reservoirs (in average 122.2) and particularly in tires (in average 109.8).

## Discussion

### Aedes albopictus and Stegomyia indices

The four Stegomyia indicators were able to detect seasonal variations of *Ae. albopictus*, as already demonstrated for *Ae. aegypti*, and highlighted the summer as the period at major risk of high mosquito density. However these indexes applied to *Ae. albopictus* showed important limitations as predictors of possible outbreaks in La Reunion, likely due to the high spatial and temporal heterogeneity values which overtake the standards for other vector-borne diseases.

Since 1996, it is classically accepted that epidemics of Yellow Fever, Dengue and other arboviruses would not occur when HI<1% and BI<5 [31]. In Cuba, BI>1 is threshold for action [23]. In Trinidad the indexes for *Ae. aegypti* during a Dengue Fever crisis between 2002 and 2004 [38], ranged from 20 to 34 during the dry season and from 30 to 46 during the wet season. In La Reunion BI for *Ae. albopictus* were always superior to 20. Similarly, HI was always superior to 12% and CI to 20%. These thresholds based on *Ae. aegypti* biology, may not be applied for a highly invasive, competitive and opportunistic species such as *Ae. albopictus*. Indeed the thresholds for defining the alert would be disease-specific depending on the transmission potential of the considered disease and of the involved mosquito species. The classical *Stegomyia* indexes revealed little use to orientate the choices of the control agency. In addition, socio-economic and environmental conditions of La Reunion can affect the ability of these indexes to characterize the variation of *Ae. albopictus* densities. Particularly, BI which was defined with *Aedes agypti* cannot be explained by the season or the meteorological data, indicating that this index is not an entomological index but more a socio-entomological index, representative of the population involvement. IRI was the less discriminant index suggesting not a great difference in human behaviour. The CI and the HI were the most discriminant index from this study, and CI is also the one (such as IRI) highlighting the HH cluster where the Chikungunya human cases appeared in 2010 (Figure S1). However an alert threshold corresponding to the biological characteristic of La Reunion remains to be defined. But the final problem concerning the prophylaxis indicators described or developed within this study remains that they are based on larval indicators, and not on adult indicators, which are the real indicators of a potential transmitted disease risk.

### Prophylaxis indicators

A better involvement of more qualified technicians, a higher number of houses visited, and a new and more efficient protocol of the control service has enhanced the quality of the collected data and the derived indexes. The global increase of all the indexes since 2007 could be explained by human behaviour with an increase of the carelessness of people since the 2006 Chikungunya epidemic. Indeed, the perception of the sanitary risk represented by *Ae. albopictus* vector decreased as well as the existing recommendations of La Reunion inhabitants (Doret et al, unpublished data). The majority of the population interviewed (79.9%) in 2011 declared no prevention of the breeding sites inside their properties contrary to 17.6% only in 2006 [36], leading to a continuous increase of Ae. albopictus, despite a good knowledge of the vectorial risk for 78.9% of them. This lack of interest impacts the BI. Some widespread behaviors (water in potted plants, regular watering) favor the persistence of breeding sites in over 22% of the controlled houses in 2011. Human behavior more than the weather can allow the maintenance of the *Ae. albopictus* population.

The most productive breeding sites are the largest ones, i.e. basins, reservoirs, and tires. Key containers are defined as individual containers of any type that contribute significantly to an urban *Aedes* problem [22]. Often, the mean number of pupae per container was considered most representative of the production of adults because of the low pupal mortality and a high correlation was reported for the number of pupae and the number of adults for *Ae. aegypti* and *Ae. albopictus* [39] [40]. In our study case, we observed no difference between pupal and larval productivity.

The productivity of breeding sites needed to introduce indexes which could take into account the real productivity depending of the type of breeding sites. These results led to the determination of a breeding site productivity ranking, defining the most productive sites needing more intervention. For the control of *Ae. albopictus* populations, we attributed a productivity coefficient for every type of breeding sites class as defined by the control vector unit. The monitoring of entomological indexes need to be simple, reproducible, reliable and pertinent. It requires the use of several indexes and a routine monitoring of adults must be developed. We propose a monitoring based on the abundance of containers and their characterization (7 classes) without counting of the number of larvae and pupae in urban breeding site. A quantifiable productivity index can help the mosquito control units to be more efficient by targeting the most productive breeding sites. This is in line with the proposition of Yebakima to define a weighted index for *Aedes* species which consider the specific productivity of each breeding site [41]. Yebakima developed a weighted Breteau Index to specifically characterize the mosquito breeding sites according to their characteristic productivity.

### Spatiotemporal heterogeneities

The mosquito population varied monthly, increasing during the summer months, according to the meteorological data. Interesting variations over the week between breeding-sites few centimeters apart were also observed not related to meteorological data, human intervention or population behaviour. The intrinsic productivity value of a breeding site is not constant despite the same environmental conditions. This random production added complexity to the analysis of the breeding site productivity and highlighted the actual limits of the monitoring. Important spatial variations of the productivity were observed from macro-scale (districts, city) to micro-scale (zones). This spatial discontinuity could be explained by the absence of ecological continuity in La Reunion, because of the alternation ravine/city and a lack of an overall/global land use. Among the 5 most productive cities, 4 are most inhabited cities and are located along the coast, at low altitudes. Properties often have small gardens with potted flowers, and high number of inhabitants increases the environmental damages with more garbage, tires and wastes leading to more mosquito breeding sites. In contrast, the 3 cities with the lowest index values are located inland at high altitudes and are isolated by high altitude geographical barriers.

In addition to this variations, an important clusterisation was found, which shows the complexity of the distribution of *Ae. albopictus* within the different localities and the difficulty of vector control. A consequence of this highly clustered, local spatial pattern is that missing some houses during vector control operations can leave intact mosquito clusters that could repopulate the area [37]. This clusterisation is also temporal as some zones can change from HH intensity to LL. This has a direct impact on vector control, preventing the localization of highly productive premises that could be targeted [37]. Because of all these variations, the data of this study can only be interpreted as a snapshot but cannot be predictive of outbreaks: the highly productive locations could not be localized in advance with this spatial approach in La Reunion.

### Rise and Weakness

We can conclude on advisedly use and misuse of these indicators. Among the good surprises, the 4 *Stegomyia* index described well the seasonal dynamic and density of the vector, even if the Breteau Index should not be considered anymore as an entomological index. These indices were fluctuant, seasonal and discriminant. We were also able to highlight some limits including that these indicators are not epidemiological risks’ indicators. Moreover, these 4 indicators are not suitable to the prediction of the epidemiological risk because the thresholds were not well defined, and the WHO thresholds were not suitable. Moreover, with the actual sampling of the control agency, the cryptic and public larvae breeding ground, tree holes or ravines’ breeding sites were not detected. And, due to the specificity of La Reunion landscape, an exhaustive sampling would imply a too high number of agents. So far, the systematic destruction of *Ae. albopictus* habitats has failed to control the species population and a continuous recolonization of areas from which individuals have been eliminated was observed. In La Reunion, the insecticide spraying and the larval breeding sites suppression were ineffective to control *Ae. albopictus* population. A specific suppression of the most productive breeding sites is likely to achieve better results, at the same time reducing the cost of the intervention.

## Supporting Information

Figure S1 **Variation of the clusterisation for each index depending of the considered yea (with Dengue and Chilungunya human cases.** This clusterisation is the Anselin representation of the clusterisation of local Moran index. HH represents High-High clusters which is a cluster with a high index surrounded with clusters with high index; LL Low-Low, LH Low-High, and HL High-Low.
(TIF)
