# Supplementary figures and images for: Spatial and Temporal Heterogeneities of Aedes albopictus Density in La Reunion Island: Rise and Weakness of Entomological Indices
Source: PLoS One. 2014 Mar 17;9(3):e91170. doi: 10.1371/journal.pone.0091170 (PMC3956670; doi:10.1371/journal.pone.0091170)

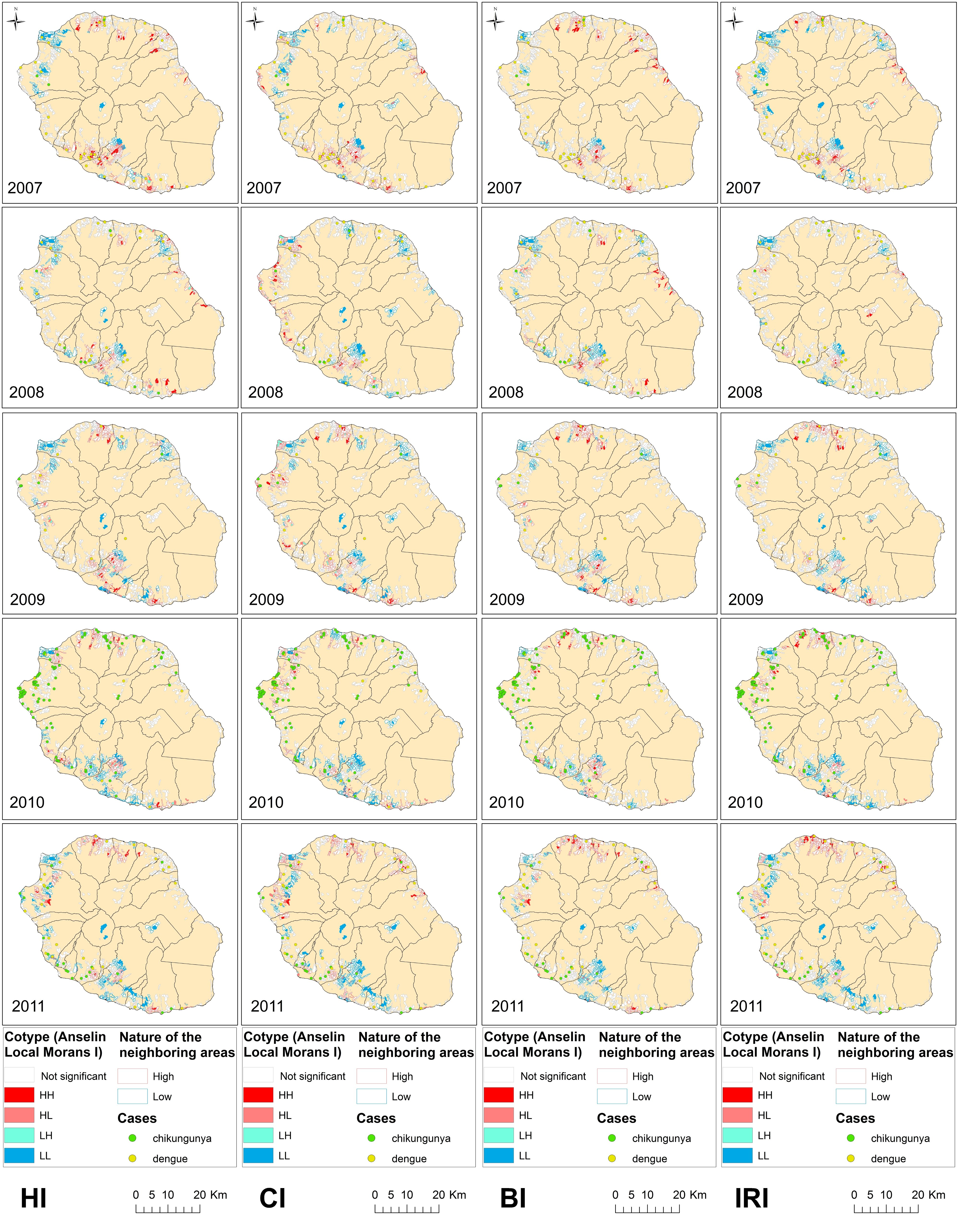

Supplement: Figure S1 — Variation of the clusterisation for each index depending of the considered yea (with Dengue and Chilungunya human cases. This clusterisation is the Anselin representation of the clusterisation of local Moran index. HH represents High-High clusters which is a cluster with a high index surrounded with clusters with high index; LL Low-Low, LH Low-High, and HL High-Low. (TIF) [file pone.0091170.s001.tif]
